# Adherence to intermittent preventive treatment for malaria in Papua New Guinean infants: A pharmacological study alongside the randomized controlled trial

**DOI:** 10.1371/journal.pone.0210789

**Published:** 2019-02-06

**Authors:** Oriane Sottas, Monia Guidi, Benjamin Thieffry, Marie Schneider, Laurent Décosterd, Ivo Mueller, Blaise Genton, Chantal Csajka, Nicolas Senn

**Affiliations:** 1 Department of Ambulatory Care and Community Medicine, Lausanne, Switzerland; 2 Service of Clinical Pharmacology, Lausanne University Hospital, Lausanne, Switzerland; 3 Clinical Pharmacy Sciences, School of Pharmaceutical Sciences, University of Geneva, University of Lausanne, Geneva, Switzerland; 4 Walter and Eliza Hall Institute, Melbourne, Australia; 5 Papua New Guinea Institute of medical research (PNG IMR), Madang, Papua New Guinea; 6 Swiss Tropical and Public Health Institute, Basel, Switzerland; Instituto Rene Rachou, BRAZIL

## Abstract

**Background:**

The intermittent preventive treatment in infants (IPTi) trial that took place in Papua New Guinea showed an overall reduction of 29% of the risk of malaria when delivering single-dose sulfadoxine-pyrimethamine (SP) associated to 3 days of amodiaquine (AQ) every three months to children during the first year of life. The aim of the present study was to assess if the last two doses of AQ were truly administered as prescribed by the parents at home based on drug level measurement and PK modelling, which is a good proxy of medication adherence. It provides also important information to discuss the efficacy of the intervention and on feasibility of self-administered preventive malaria treatment.

**Methods and findings:**

During the three-arm randomized double-blinded IPTi trial, each child was prescribed one dose of SP (day 0) and 3 doses of either AQ or artesunate (AS) at day 0, 1 & 2 adjusted to weight or placebo. Treatments were given at 3, 6, 9 and 12 months of age. The first day of treatment was delivered by nursing staff (initiation under directly observed treatment (DOT)) and the two last doses of AQ or AS by parents at home without supervision. For this cross-sectional study, 206 consecutive children already involved in the IPTi trial were enrolled over a 2-month period. At the time of the survey, allocation of the children to one of the three arms was not known. Blood samples for drug level measurement were collected from finger pricks one day after the planned last third dose intake. Only children allocated to the SP-AQ arm were included in the present analysis. Indeed, the half-life of AS is too short to assess if drugs were given on not. Because of the short half-life of AQ, desethyl-AQ (metabolite of AQ (DAQ)) measurements were used to investigate AQ medication adherence. Two PK (PK) models from previously published studies in paediatric populations were applied to the dataset using non-linear mixed effect modelling (NONMEM) to estimate the number of doses really given by the parents. The study nurse reported the administration time for the first AQ dose while it was estimated by the parents for the remaining two doses. Out of 206 children, 64 were in the SP-AQ arm. The adjusted dosing history for each individual was identified as the one with the lowest difference between observed and individual predicted concentrations estimated by the two PK models for all the possible adherence schemes. The median (range) blood concentration AQ in AQ arm was 9.3 ng/mL (0–1427.8 ng/mL), (Quartiles 1–3: 2.4 ng/mL -22.2 ng/mL). The median (range) for DAQ was 162.0 ng/mL (0–712 ng/mL), (Quartiles 1–3: 80.4 ng/mL-267.7 ng/mL). Under the assumption of full adherence for all participants, a marked underprediction of concentrations was observed using both PK models. Our results suggest that only 39–50% of children received the three scheduled doses of AQ as prescribed, 33–37% two doses and 17–24% received only the first dose administered by the study nurse. Both models were highly congruent to classify adherence patterns.

**Conclusions:**

Considering the IPTi intervention, our results seem to indicate that medication adherence is low in the ideal trial research setting and is likely to be even lower if given in day-to-day practice, questioning the real impact that this intervention might have. More generally, the estimation of the number of doses truly administered, a proxy measure of adherence and an assessment of the feasibility of the mode of administration, should be more thoroughly studied when discussing the efficacy of the interventions in trials investigating self-administered malaria preventive treatments.

## Introduction

In Papua New Guinea (PNG), malaria transmission is high and the disease is responsible for several thousands of deaths each year. The number of estimated cases in 2013 was between 800’000 and 2’000’000[1]. The major plasmodium species present in PNG are *Plasmodium falciparum* and *Plasmodium vivax*.

Intermittent preventive treatment in infants (IPTi) aims to reduce malaria risk by delivering several antimalarial drugs, three or four times, coinciding with immunization planned by the Expended Program on Immunization (EPI) to children younger than 1 year[1–3], particularly in regions where malaria transmission is moderate to high. Most of the drugs proposed for this intervention have a long half-life, such as sulfadoxine-pyrimethamine (SP)[1]. The principal aim of the IPTi trial that took place in Papua New Guinea was to investigate the preventive efficacy and the overall risk reduction against falciparum and vivax malaria of delivering four courses of antimalarial drugs to children during the first year of life. This three-arm study, completed in 2010, showed an overall reduction of 29% in malaria risk using SP associated with amodiaquine (AQ) compared to placebo[4] and no significant effect of SP associated to with artesunate (AS) compared to placebo.

As the parents at home directly administer the second and third treatment doses without supervision, overall the number of doses truly administered remains largely uncertain, which might have a direct impact on the efficacy of the intervention. From a perspective of implementing IPTi, as with any therapy with self-administered treatment, knowing the adherence level is a key element. There are several reasons why infants might not receive the correct amount of drugs: nurses not giving the right dosage or crushing the pill, which might induce some loss of the active ingredient, children vomiting the drugs or the parents not administering drugs at home. The latter reason might be especially common in the case of IPTi, as it is a preventive intervention and parents might find it less useful or might prefer keeping the drugs for later use in case of malaria-like symptoms onset. In a qualitative study performed in children of the present IPTI trial, about 15% of the mothers mentioned not having given the drugs according to instructions[5]. In another qualitative study realised in five African countries by Gysels *et al.[5]*, they reported that it was common for mothers to forget giving the tablets or keep them for later use.

Medication adherence is defined as the process by which patients take their medications as prescribed by their healthcare providers[6]. Medication adherence is composed of three core components: initiation, implementation (dosing and daily timing) and discontinuation. Time between first and last dose is defined as “persistence”. Assessment of medication adherence uses direct and indirect methods[7]. Indirect methods (e.g. self-report questionnaires) tend to overestimate real medication adherence. Direct methods, whenever available, such as drug blood-level monitoring, represent more objective assessments. However, concentration measurements do not reflect long-term adherence to the treatment and important inter-individual (e.g. genetic, age, body weight, concomitant drugs, foods, disease) and intra-individual variations (e.g. random changes in concentrations from one visit to the other) in drug levels occur, which make the measurement of adherence more complex. We suggest to use PK (PK) modelling to estimate the number of doses truly taken according to blood drug concentrations at a defined time point and taking into consideration other factors related to the patient (weight, age…).This requires expertise in drug modelling and availability of reliable PK models. To our knowledge, PK has never been used to test adherence to malaria preventive treatments.

The aim of the present study was to assess if the last two doses of AQ were truly administered as prescribed by the parents at home based on drug level measurement and PK modelling, which is a good proxy of medication adherence and an assessment of the feasibility of self-administred malaria preventive treatments. It provides also important information to discuss the efficacy of the intervention. It was performed in a random sample of 206 study participants included in the randomized controlled trial performed in Papua New Guinea.

## Methods

The IPTi randomized controlled trial was conducted in PNG in the Mugil area of the Sumkar District, Madang Province, between June 2006 and May 2010 and included 1125 children. Each study participant received four courses of treatment at 3, 6, 9 and 12 months. Each course consisted of three days of treatment and each time, nursing staff delivered SP (single dose) and the first dose of AQ or AS (day 0, D0) by initiation under directly observed treatment (DOT) and parents at home without supervision the others doses of AQ or AS (day 1 and 2). The tablets were given with sweet syrup after being crushed by the nurse or parents. The first drug dose (D0) was given a second time if the child vomited within 30 minutes. A community reporter living in the village was in charge of visiting the children to check that no adverse events occurred. He/she had no responsibility for giving drugs or supervising drug intake. All details concerning the trial and efficacy results have been published elsewhere[5]. The study was carried out in accordance with Good Clinical Practice (ICH GCP E6) guidelines and externally monitored by two independent monitors and the Data Safety Monitoring Board. The study (main protocol and present amendment) was approved by the PNG Medical Research Advisory Committee (MRAC number 05.20). The trial was registered on http://www.clinicaltrials.gov (number NCT00285662) and formed part of the IPTi Consortium (http://www.ipti-malaria.org). In concordance with MRAC regulations, written informed consent was obtained from the parents both for the main IPTi study and the present work.

For the present cross-sectional study, 206 consecutive children already part of the IPTi trial, were enrolled over an approximate 2-month period. At the time of sampling, allocation groups in the IPTi trial were unknown. Indeed, patients were assigned blindly into one of the three trial arms (SP-AQ, SP-AS and placebo), so we enrolled a large number of children aiming to have approximately 60 patients in each arm.

**Fig 1:**
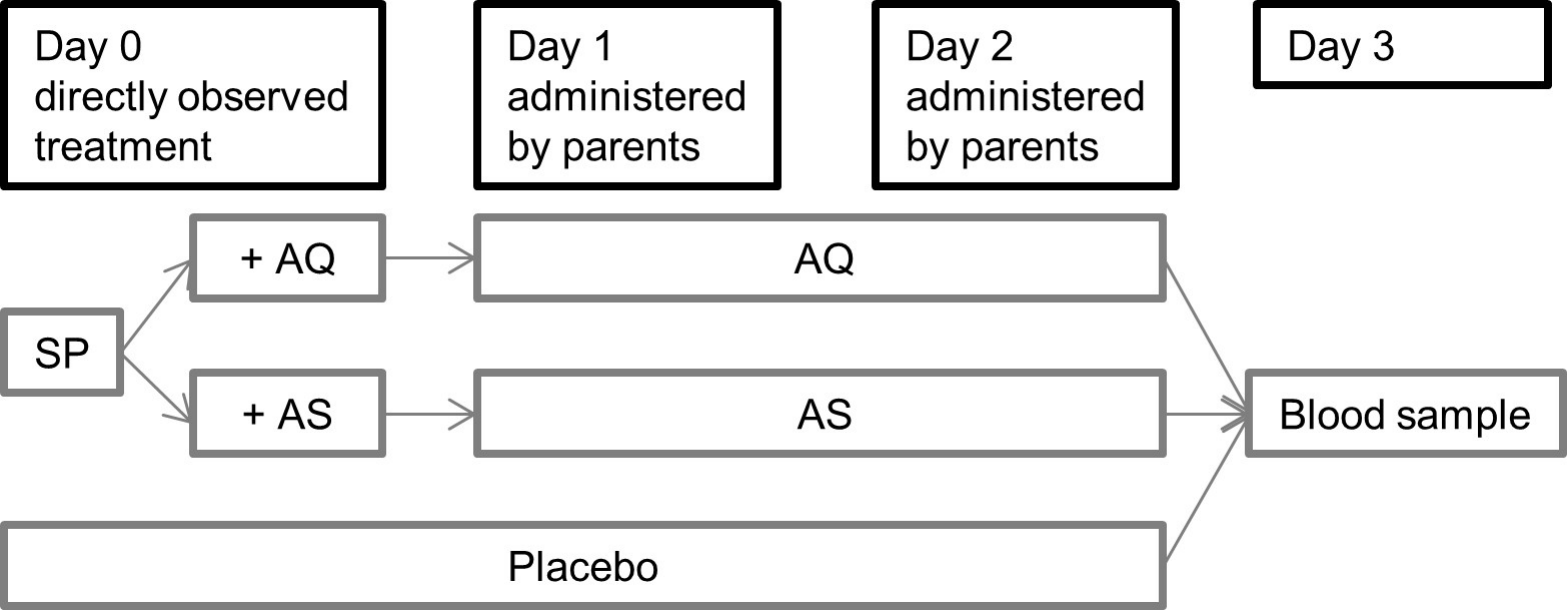
Diagram of the course of the study.

Nurses of the study team visited each of the 206 study participants at home one day (D3) after the theoretical last-dose taken (D2). To avoid biasing the results of the study, parents were not informed in advance of the visit. Blood samples for drug level measurement were collected from finger pricks. The course of the study is detailed in **Fig 1**. An experienced study nurse also collected at home parents’ estimated time of drug administration on day 1 and day 2 using a scale that divides day-time into 2-hour slices. Parents specifically consented to this additional blood collection, not included in the main trial.

The blood samples of the 206 participants were used to measure the levels of IPTi drugs (SP, AS and AQ + AQ-metabolites). Drug level measurements were conducted at the laboratory of the Division of clinical pharmacology of the University hospital of Lausanne, Switzerland, using a chromatography-tandem mass spectrometry method (LC-MS/MS) (Hodel-EM et al 2009).

Children included in the PK analysis are those whose parents reported having given drugs and with drug levels available. Each study participant was at a different stage of the study and thus received the treatment course at 3, 6, 9 or 12 months. We performed the analysis on patients in the AQ arm only. More specifically, we used desethyl-amodiaquine (metabolite of AQ (DAQ)) measurements to if drugs were truly administered by parents as AS and AQ concentrations are low or undetectable one day after drug administration because of their short half-lives (20–45 minutes and 4 hours in adults, respectively[8, 9]. This approach provides a good proxy for medication adherence and the feasibility of self-administration of malaria preventive treatments in infants. For clarity, we will refer to the term “medication adherence” also we are fully aware that it reflects only how many of the two last doses were really given by the parents in the specific frame of the trial. It the perspective of the trial, it provides also valuable information to discuss the efficacy of the IPTi intervention. SP was not investigated for adherence as the study nurses administered the single dose. Children who were ill (criteria for malaria were: fever and a positive rapid diagnostic test for malaria) up to one week before the preventive treatment was due did not receive study drugs but received curative treatment with lumefantrine-arthemeter (Coartem) and therefore were excluded from the study.

Non-linear mixed effect modelling (NONMEM, v7.3.0, ICON development Solutions, Ellicott City, MD, USA)) was employed to predict individual DAQ concentration (NONMEM option MAXVAL = 0) [10]. This program offers the opportunity to characterize concentration-time profiles of drugs by estimating individual and population PK parameters, assessing their variability and quantifying the impact of subjects’ factors on the P parameters. Since dosing history was unknown and evaluated in the present study, we decided to apply previously published models to our data instead of conducting a population PK analysis. A literature research was performed with the aim of identifying PK models of AQ and DAQ disposition in a paediatric population similar to the investigated one. We retrieved the mean PK parameters with their variability and applied such models to our dataset to predict DAQ concentration under the assumption of full (three doses, *i*.*e*. planned dosing history) or partial (only the dose on day 0, doses on days 0 and 1, or doses on days 0 and 2) medication adherence. We calculated individual residuals (IRES) as the difference between predicted (C_pred_) and (C_obs_) observed concentrations in each of the four adherence schemes and identified the true dosing history for each child as the one associated with the lowest IRES value among the four possible adherence patterns. Children were thus categorized into groups according to the identified dosing history: subjects receiving a dose on day 0 (defined as configuration of adherence D_1, *i*.*e*. only the first dose was taken), on days 0 and 1 (D_12) on days 0 and 2 (D_13), and those who completed the treatment (D_123). We estimated bias and precision of the model assuming theoretical full adherence and the model-corrected dosage history according to IRES values by mean prediction error ($\mathrm{MPE}=\frac{\sum C_{\mathrm{pred}}-C_{\mathrm{obs}}}{N}$, where C_pred_ and C_obs_ as previously defined and N the number of observations) with the associated CI_95%_, and root mean squared error (RMSE = $\sqrt{\frac{\sum {\left(C_{\mathrm{pred}}-C_{\mathrm{obs}}\right)}^{2}}{N}}$) [11]. R v3.2.3 (R Development Core Team, http://www.r-project.org/) was used for statistical analyses and figures generation.

## Results

### Literature search

A Pubmed search allowed us to identify two previously published studies in the paediatric population. Hietala *et al*. [8] analysed data collected from children aged from 3 months to 12 years from Tanzania and Papua New Guinea, while Stepniewska *et al*. [12] analysed a population of children from 6 months to 5 years in Burkina Faso. In both studies, DAQ PKs was best described by a two-compartment model with first-order absorption and elimination. Body weight was found to influence DAQ disposition. An additional association between age and DAQ clearance was reported by Hietala *et al.[8]*. As described in **Table 1**, both models provide similar PK parameters. For both models, a very long DAQ terminal half-life (from 3 to 8 days in Hietala *et al*. [8] and from 7 to 12 days in Stepniewska *et al*. [12]) was estimated.

**Table 1:** Estimated PK parameters for DAQ, for both models.

	Models	
Parameters	Hietala(10)	Stepniewska(12)
	Estimate (RSE%)	Estimate (SE)
**Ka (h**^**-1**^**)**	0.13 (31)	0.13 (fixed)
**CL/F (L Kg**^**-1**^ **h**^**-1**^**)**	0.67 (10)	0.610 (0.038)
**Q (L Kg**^**-1**^ **h**^**-1**^**)**	1.3 (23)	0.680 (0.306)
**V**_**central**_**/F (L Kg**^**-1**^**)**	12.8 (44)	35.4 (11.5)
**V**_**peripheral**_**/F (L Kg**^**-1**^**)**	62.4 (9)	87.9 (17.1)
**T ½ (days)**	4.9 [3.3–8.0]^1^	9.0 [7.3–11.6]^1^
**σ**	25 fixed	0.457

Ka: absorption rate constant; CL/F: total clearance; Q: intercompartmental clearance; V_central_/F: volume of the central compartment; V_peripheral_/F: volume of the peripheral compartment; F: fraction of drug absorbed; σ: residual additive error on the log_e_ scale

^1^range of predicted values

### Pharmacokinetic analyses

**Fig 2:**
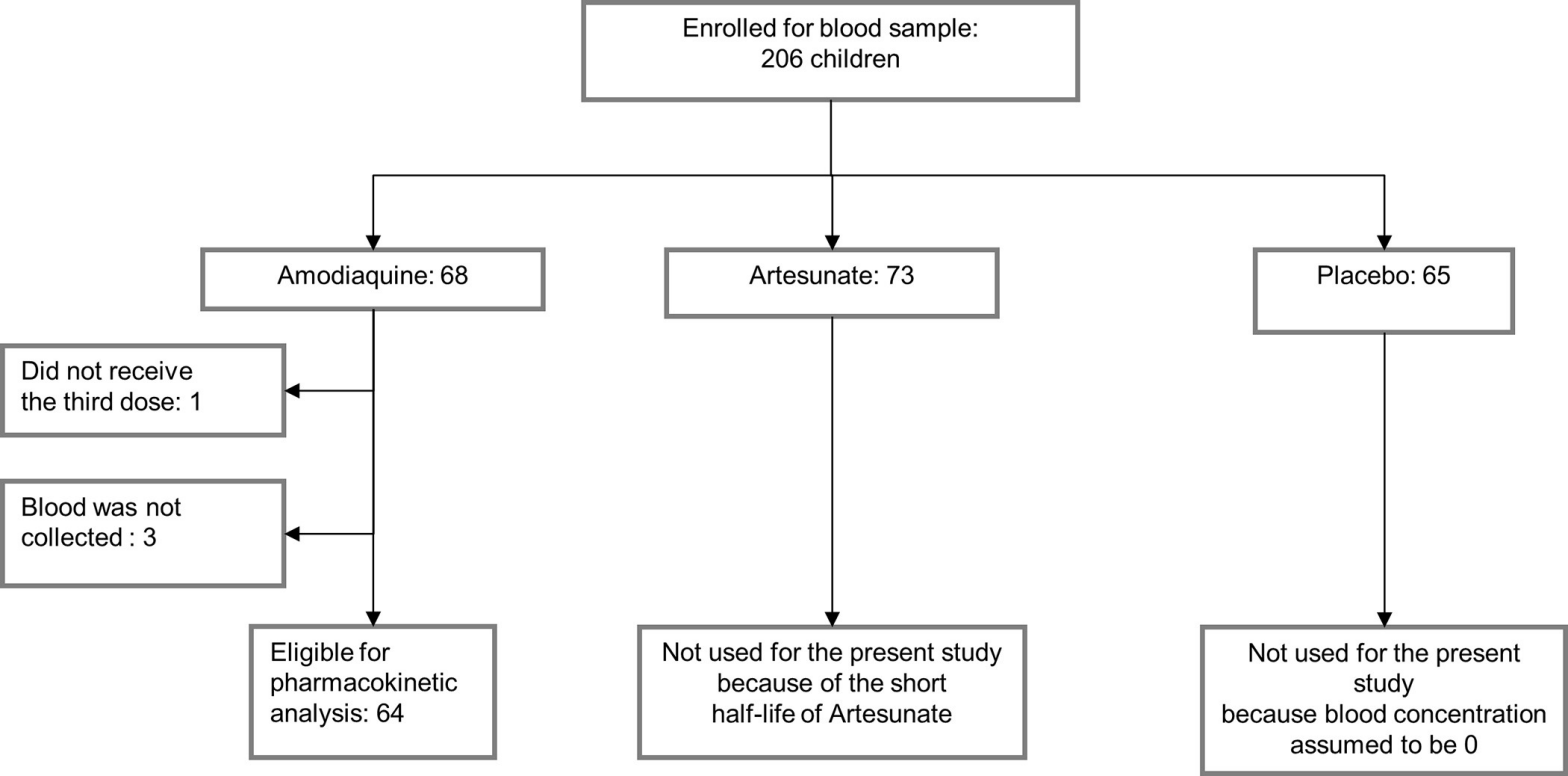
Selection of the patients eligible for the PK analysis.

Out of 206 children, 68 belonged to the SP-AQ arm. One was removed because parents said they did not give the third dose and three because blood was not collected, thus 64 children fulfilled the inclusion criteria (**Fig 2**). Age, weight and gender characteristics of the study participants are described in **Table 2** and compared to those of patients in both studies used as models for the PK analysis.

**Table 2:** Characteristics of the patients in the present study compared to both models used.

	Present study n = 64	Hietala[10] n = 329	Stepniewska[12] n = 70
Age (years or months) (mean[range])	6 months [2–13]	2.5 years [0.3–10]	27.5 months [7–59]
Weight (kg)	7 [4–10]	13.15^1^ [8–27]	12 [6–31]
Gender ratio (male/female)	43/21	166/163	36/34

^*1*^n = 117, weight was not specified for all patients

The median (range) AQ blood concentration in the AQ arm was 9.3 ng/mL (0–1427.8 ng/mL), (Quartiles 1–3: 2.4 ng/mL -22.2 ng/mL). The median (range) for DAQ was 162.0 ng/mL (0–712 ng/mL), (Quartiles 1–3: 80.4 ng/mL-267.7 ng/mL).

In comparison, the median (range) AQ blood concentration in the placebo arm was 0 ng/mL (0–137.0 ng/mL), (Quartiles 1–3: 0 ng/mL -0.2 ng/mL). The median (range) for DAQ was 0.7 ng/mL (0–202.1 ng/mL), (Quartiles 1–3: 0 ng/mL-6.3 ng/mL). Overall, 16/65 patients had detectable concentrations of AQ and 34/65 of DAQ.

**Fig 3:**
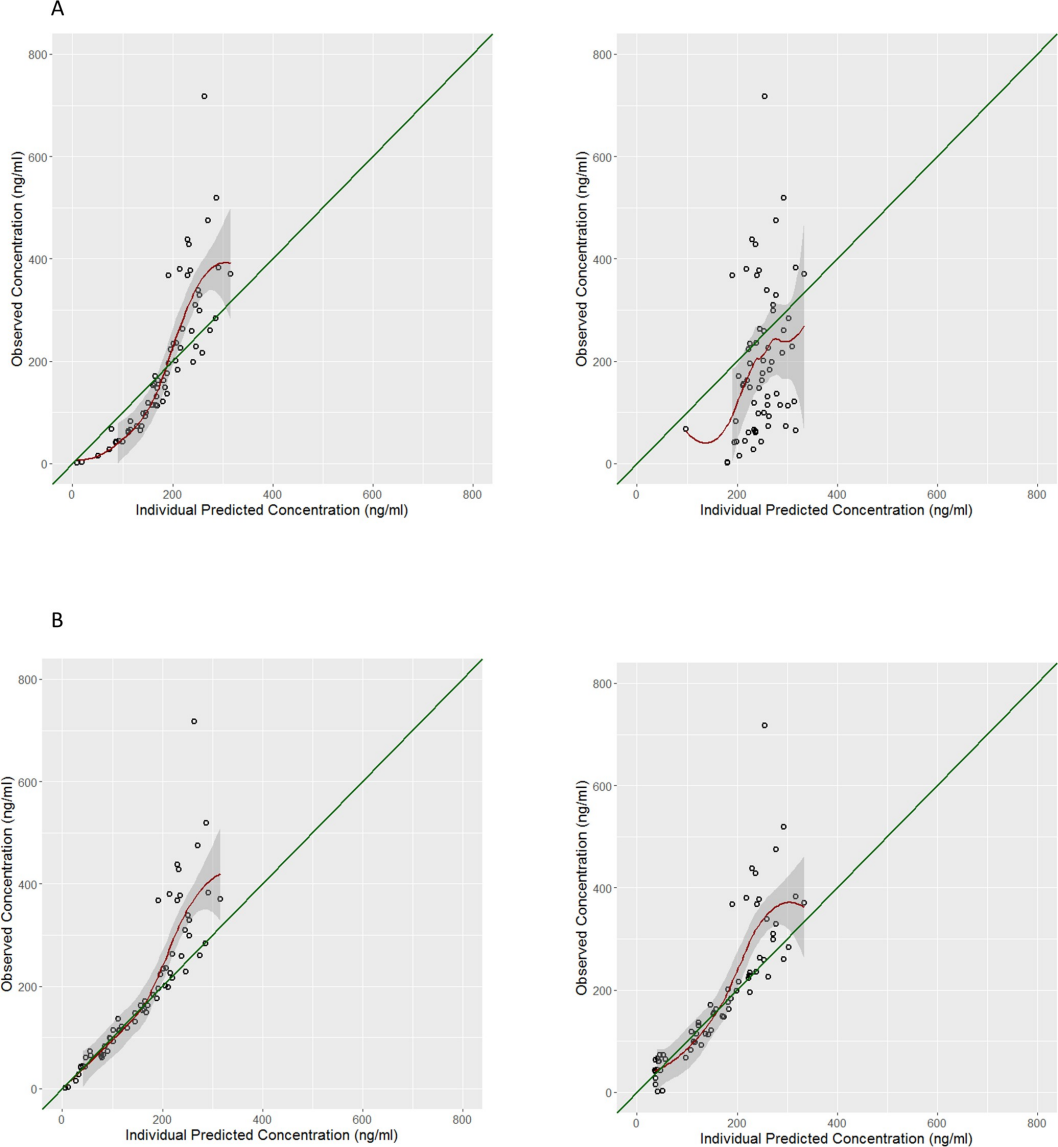
Observed versus individual predicted DAQ concentrations under the assumption of full and model-identified adherence.

Assuming full adherence, we observed a marked under prediction of concentrations using both the Hietala and the Stepniewska models. **Fig 3** illustrates the observed vs. individual Hietala and Stepniewska model-predicted concentrations for the theoretical and adjusted dosing datasets.

The results suggest that 11 children (17%) are in adherence configuration D_1, 8 (13%) in D_13, 13 (20%) in D_12 and 32 (50%) received full treatment according to the Hietala model. Applying the Stepniewska model provided similar results, with 15 children (24%) in configuration D_1, 13 (20%) in D_13, 11 (17%) in D_12 and 25 (39%) received all three doses. The two models agreed on the adherence schemes for 80% of the children. Discrepant results were observed in thirteen children. For seven of them, the adherence schemes were D_123 vs. D_13, for four of them, D_12 vs. D_1, and for two of them, D_13 vs. D_12, comparing the Hietala vs. Stepniewska models, respectively.

The bias (95% confidence interval CI_95%_) was 20% (CI_95%_ 4–38%) with a precision of 79% for the Hietala model and was 82% (CI_95%_ 40–138%) with a precision of 236% using the Stepniewska model. Under the assumption of model-identified adherence, the initial bias and precision of the Hietala model decreased to -4% (CI_95%_ -14-6%) with a precision of 50% and to 2% (CI_95%_ -14-20%) with a precision of 97% using the Stepniewska model and was thus not significantly different from zero. The observed *vs*. individual predicted concentrations under the assumption of each adherence configuration for both models are shown in S1 and S2 Figs.

**Tables 3 and 4** show the four adherence patterns, stratified by age, according to both models.

**Table 3:** Results by age according to Hietala model.

	Adjusted dosing dataset				
Course of treatment	Dose 1	Doses 1 and 3	Doses 1 and 2	Doses 1, 2 and 3	Nb of patients
**1 at 3 months**	4 (17%)	3 (13%)	3 (13%)	13 (57%)	23
**(Nb of children (% by age))**					
**2 at 6 months**	2 (13%)	3 (19%)	3 (19%)	8 (50%)	16
**(Nb of children (% by age))**					
**3 at 9 months**	3 (23%)	1 (8%)	2 (15%)	7 (54%)	13
**(Nb of children (% by age))**					
**4 at 12 months**	2 (17%)	1 (8%)	5 (42%)	4 (33%)	12
**(Nb of children (% by age))**					
**Total**	**11 (17%)**	**8 (13%)**	**13 (20%)**	**32 (50%)**	**64**
**(Nb of children (% of the total))**					

**Table 4:** Results by age according to Stepniewska model. Nb children (% by age).

	Adjusted dosing dataset				
Course of treatment	Dose 1	Doses 1 and 3	Doses 1 and 2	Doses 1, 2 and 3	Nb of patients
**1 at 3 months**	4 (17%)	5 (22%)	4 (17%)	10 (44%)	23
**(Nb of children (% by age))**					
**2 at 6 months**	3 (19%)	5 (31%)	2 (13%)	6 (37%)	16
**(Nb of children (% by age))**					
**3 at 9 months**	4 (31%)	1 (8%)	2 (15%)	6 (46%)	13
**(Nb of children (% by age))**					
**4 at 12 months**	4 (33%)	2 (17%)	3 (25%)	3 (25%)	12
**(Nb of children (% by age))**					
**Total**	**15 (24%)**	**13 (20%)**	**11 (17%)**	**25 (39%)**	**64**
**(Nb of children (% of the total))**					

## Discussion

This study suggests that a majority of children did not receive the full IPTi treatment within the frame of the randomized trial, which could substantially decrease the optimal efficacy of the intervention. Our results indeed showed that only 39 to 50% of children, depending on the PK model used, received the three scheduled doses of AQ, while 33 to 37% received two doses and 17 to 24% received only the first dose, administered by the study nurse.

In the present study, drug concentration measurements was used as a proxy of medication adherence in the frame of the trial. Even though it is not a direct measure of adherence, we believe that this approach provides important information related to adherence in the context of a trial that might also useful, by extension, to estimate what would happen if the intervention would be implemented in real life. It informs also on the feasibility of the self-administration approach for preventive malaria treatments in infants. Finally, this information is also of prime importance to discuss the overall efficacy of the intervention.

Results of the modelling showed that imputing a partial adherence instead of considering a full dosing schedule explained the discrepancy between measured and expected drug concentrations. The major bias observed while assuming full adherence to prescribed dosing became not-significantly different from 0 using the adjusted dosing history. This supports the adequacy of the actual dosing history including several scenarios of missed doses. Moreover, although some differences in PK estimates were present between both models, they were highly congruent at classifying adherence patterns in children, which reinforces the validity the results. We can therefore be confident that this approach provides a reliable estimation of short-term AQ medication adherence.

We observed different medication adherence patterns. Adherence to the first dose was 100%, what is reinsuring about the study procedure, as it was DOT-delivered by the study nurse. Only half of the children showed adherence to the subsequent doses, while the others had only partial medication adherence. Either they did not take the last dose, and therefore discontinued IPTi too early, or they did not take the middle dose. Early discontinuation was more frequent (37–41%) than non-ingestion of the middle dose (13–20%). It is interesting to note that adherence to the full IPTI treatment in this study was very close to the levels found in the literature for real life adherence to long-term medication for chronic diseases (around 50%), a totally different context[13].

These results show that, even in the frame of a well-controlled clinical trial, adherence is far from optimal. Furthermore,Even if parents at home were responsible to administer the last two doses, a community reporter was responsible for visiting families each treatment day, checking for serious adverse events and collecting empty drug bags (thus serving indirectly as a reminder for drug intake). This type of check procedure is possible only in the frame of a trial, and certainly not in routine care. Although impossible to make clear assumptions one way or another, we may still wonder what the real efficacy of SP-AQ would have been if the adherence had been 100% instead of 50%.

The results of this study also question the potential impact of a preventive intervention with self-administered treatment such as IPTi if implemented in a real-life context. Indeed, the observed adherence of 37% to 50% for the full treatment course is certainly the highest that one could expect in a less controlled context. Furthermore, for an intervention such as IPTi, most of the protection is due to the prophylactic effect long-acting drugs as mentioned in the main trial publication. [4] SP is therefore likely to be responsible for the most important part of the observed protection, and the results of this study reinforces the hypothesis that the added benefit of AQ is limited, also if it has also a relative long half-life (> 7days). In the trial, adherence to SP was close to 100%, which is unachievable in daily practice, even if the dose is given by a health professional. In an observational implementation-effectiveness study in Tanzania conducted by Armstrong Schellenberg *et al*., it was observed that only 47 to 76% children received the first dose of SP in an area where IPTi is carried out. They also assumed that children without health cards did not receive IPTi and approximately one third did not receive IPTi at the same time as immunization as staff forgot to give the drug or it was out-of-stock[10]. Thus, it is likely that, if IPTi is widely implemented in PNG, the overall reduction of malaria risk would be much lower than the 29% observed.

On the other hand, one might argue that the overall risk reduction observed is under-estimated due to the fact that many children take non-prescribed “over-the-counter” antimalarial drugs, especially AQ. In the present study, even if the measures of AQ blood concentrations revealed that half of the children in the placebo arm took “over-the-counter” AQ and almost all SP (data not shown), the concentrations were much lower than in the SP-AQ arm. It is likely therefore that SP and AQ “over-the-counter” medications had only a marginal effect on the overall protection in this study population even more as resistance to these two drugs were low at the time of the study.

The reasons for only 50% or less of the children receiving all three doses of IPTi may be multiple. First, it could be related to the preventive nature of the intervention. Indeed, parents may decide to keep the antimalarial drugs for later use, in case of illness rather than giving it to their healthy child. Second, since AQ tastes very bitter, children might have vomited or spat out the drugs. Third, and most probably, parents may just have forgotten to give the treatment, especially as no immediate benefit of the treatment is expected. Lastly, almost half the children aged 3-months received the three doses while only one third of those aged 12 months did. These results seem to indicate that parents are more likely to give a preventive treatment to younger children than to older ones. Overall, the results of the study point out that self-administration of malaria preventive treatments is probably a suboptimal approach.

More generally, our study showed that it is feasible and reliable to assess objectively malaria treatment adherence using PK modelling, even in complex research settings. We are not aware of other studies that use this approach to investigate malaria adherence in developing, at least for preventive treatments. Few studies have compared drugs levels at the end of curative treatment courses to make broad assumptions on adherence, but not to measure precisely the number of doses taken.[14, 15] Our study highlights also the important gap between self-reported adherence (all parents reported having given the full treatment course) and an objective assessment, which is of importance. Furthermore, for the present trial, a qualitative acceptability study was conducted and concluded that the intervention was well suited to the local PNG culture.[5] This illustrates how difficult it is to make clear assumptions on the true adherence trough other ways than objective drug level measurements and PK analysis. Thus, this study provides a new insight on how to assess objectively the true adherence to short courses of malaria treatments. This can have important implications for researchers and policy makers, who could integrate more often such approaches at the time of interpreting effect sizes of interventions and allow a better anticipation of the potential benefits that an intervention might have. This could also apply to other research filed than malaria.

A first limitation is that a full PK model could not be built due to the scarcity of data and we performed the analysis based on previously published models in children. These models were derived from older children, mostly from Africa, and were not validated in younger children in PNG. However, in order to control for an “effect model”, we tested both published models and found very similar results, which reinforces the reliability of the results. Second, the relatively small sample size of the study might not reflect the adherence of the entire cohort of patients. On the other hand, even if some uncertainties may remain on the exact proportion of patients with poor adherence, the objective of this study was more to provide estimations rather than an exact figure. Furthermore, this is probably a conservative estimate of the adherence, mainly because we cannot exclude that some parents may have administered the treatment to their child at last minute because they heard that study staff were coming (even if our visit was not announced). Lastly, some uncertainties exist about the exact time at which the drugs were taken. However, the careful history taking performed by the nurses allowed a time estimate of around 2 hours, which is sufficient for the purpose of this analysis, especially considering the long half-life of DAQ.

## Conclusion

This study showed that less than half of the study participants in an IPTi randomized controlled clinical trial took the full three-day course of antimalarial preventive medication. This is very low considering that the first dose was directly administered by a study nurse and the study was closely monitored. These results seem to indicate that medication adherence is low in an ideal research setting and therefore might be even lower if implemented in day-to-day practice, questioning the impact that this intervention might really have. It also reinforces the hypothesis that AQ add little to the overall efficacy of the IPTi intervention, which relies mostly on the long lasting effect of a single dose of SP. Finally, these results question the self-administration approach for preventive malaria treatments in children. This is relevant information, not only for the specific IPTi intervention, but also more generally for drug trials of this kind. Indeed, researchers should consider using this approach when designing new studies and estimating effect sizes of short malaria treatment courses.

## Supporting information

S1 Fig Observed vs model-predicted concentrations by Hietala et al. (9) under the four tested adherence patterns. (DOCX)

S2 Fig Observed vs model-predicted concentrations by Stepniewska et al. (11) under the four tested adherence patterns. (DOCX)

## Abbreviations

AQ: amodiaquine
DAQ: desethyl-amodiaquine
DOT: directly observed treatment
EPI: Expended Program on Immunization
IPTI: Intermittent preventive treatment in infants
IRES: individual residuals
MPE: mean prediction error
NONMEM: non-linear mixed effect modelling
PK: PK
PNG: Papua New Guinea
RMSE: root mean squared error
SP: sulfadoxine-pyrimethamine
