# Supplementary material for: Adherence to intermittent preventive treatment for malaria in Papua New Guinean infants: A pharmacological study alongside the randomized controlled trial
Source: PLoS One. 2019 Feb 6;14(2):e0210789. doi: 10.1371/journal.pone.0210789 (PMC6364960; doi:10.1371/journal.pone.0210789)
Supplement: S2 Fig — (DOCX) [file pone.0210789.s002.docx]

**S2 Fig:** Observed vs model-predicted concentrations by Stepniewska *et al.* (11) under the four tested adherence patterns (full adherence to the treatment (D_123), amodiaquine only on days 0 and 1 (D_12), on days 0 and 2 (D_13) or on day 1 (D_1)). The red dots represent the patients that were effectively assigned to the tested group, the black dots those assigned to alternative dosing history group. Loess (locally estimated scatterplot smoothing) curves (dark-red) with 95% confidence intervals (shaded grey surfaces) and identity lines (dark-green) are shown.


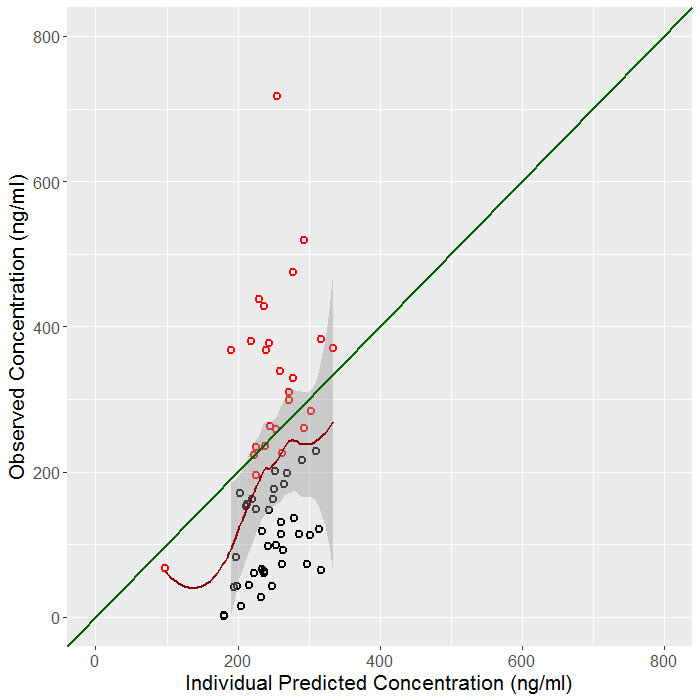

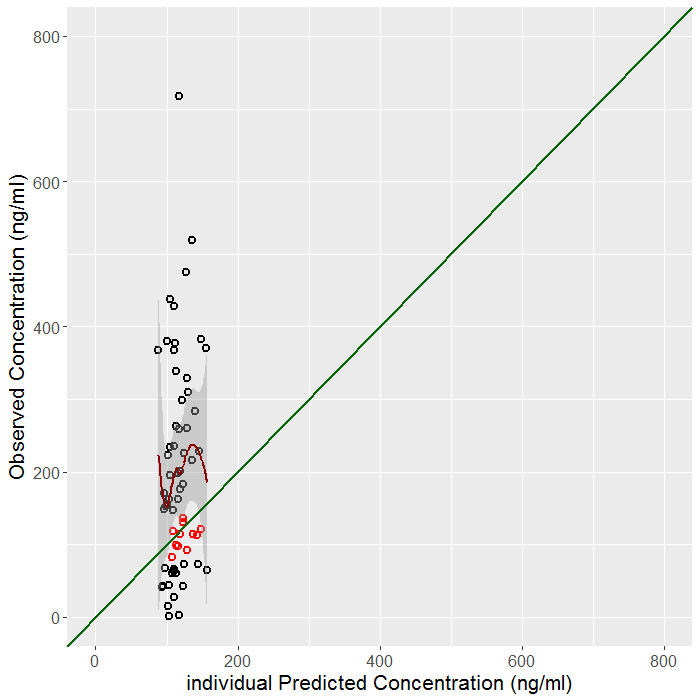

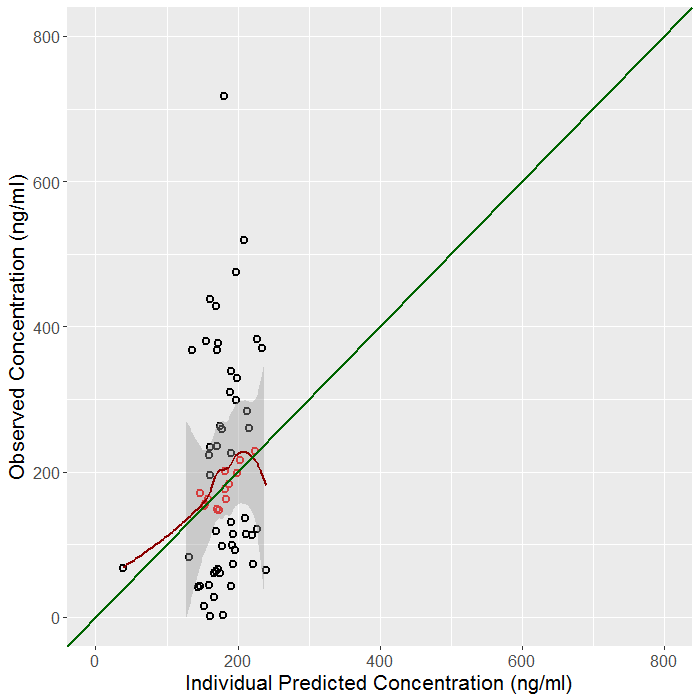

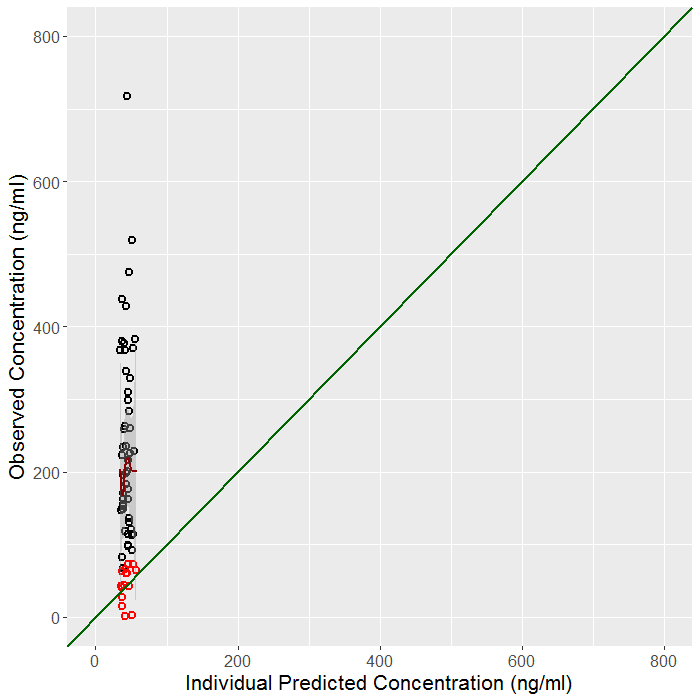


**D_1**

**D_13**

**D_12**

**D_123**
